# Nonlinear Inverse Analysis for Predicting the Tensile Properties of Strain-Softening and Strain-Hardening UHPFRC

**DOI:** 10.3390/ma15093067

**Published:** 2022-04-22

**Authors:** Yi-Qing Guo, Jun-Yan Wang, Jin-Ben Gu

**Affiliations:** 1 Key Laboratory of Advanced Civil Engineering Materials, Ministry of Education, Tongji University, Shanghai 201804, China; guoyiqing@tongji.edu.cn (Y.-Q.G.); jb.gu@tongji.edu.cn (J.-B.G.); 2 Department of Structural Engineering, Tongji University, Shanghai 201804, China

**Keywords:** fiber-reinforced cement composites (FRCC), ultra-high-performance fiber-reinforced concrete (UHPFRC), bending test, tensile behavior, load–deflection response, inverse analysis

## Abstract

The tensile stress–strain response is considered to be the most important and fundamental mechanical property of ultra-high-performance fiber-reinforced concrete (UHPFRC). Nevertheless, it is still a challenging matter for researchers to determine the tensile properties of UHPFRC. As a simpler alternative to the direct tensile test, bending tests are widely performed to characterize the tensile behavior of UHPFRC, but require further consideration and a sophisticated inverse analysis procedure. In order to efficiently predict the tensile properties of UHPFRC, a nonlinear inverse method based on notched three-point bending tests (3PBT) was proposed in this paper. A total of fifteen UHPFRC beams were fabricated and tested to evaluate the sensitivity of the predicted tensile behavior to variations in fiber volume fraction. A segmented stress–strain model was used, which is capable of describing the various tensile properties of UHPFRC, including strain softening and strain hardening. A more approximate formulation was adopted to simulate the load–deflection response of UHPFRC beam specimens. The closed-form analytical solutions were validated by tensile test results and existing methods in literature. Finally, parametric studies were also conducted to investigate the robustness of the proposed method. The load–deflection responses obtained from notched 3PBT could be easily converted into tensile properties with this inverse method.

## 1. Introduction

Ultra-high-performance fiber-reinforced concrete (UHPFRC) is today widely used in structural applications, such as civil infrastructures [1,2], prefabricated components [3,4], tunnel linings [5], retaining walls [6], and existing structure repairs [7]. According to the classification of the fiber-reinforced cement composite (FRCC) [8], UHPFRC can be considered as a special type of FRCC, which is characterized by an optimized gradation of granular constituents along with a low water-to-binder ratio that results in a high durability and a high percentage of discontinuous internal micro-fine steel fiber reinforcement providing a hardened concrete with excellent ductility [9,10]. Due to modified mechanical properties, including durability, ductility, and crack width control capacity, UHPFRC can be used for reducing the deadweight of the superstructure, increasing the span capability of the bridge, and saving on the maintenance cost during the service life [11].

**Figure 1:**
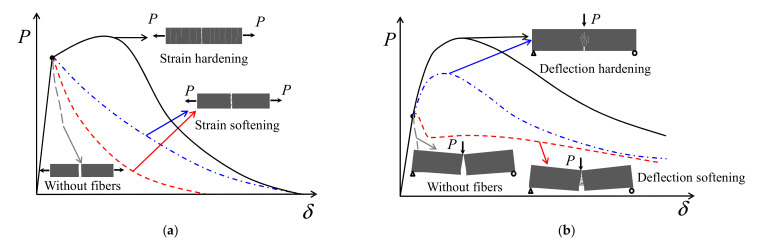
Schematic description of: (**a**) Softening and hardening behavior of UHPFRC in uniaxial tension; (**b**) Corresponding load–deflection response under three point bending.

Generally, structural design with UHPFRC requires a reliable index about the tensile performance, which is considered to be the most important property of this advanced material [12]. However, the mechanical response of UHPFRC in tension is significantly influenced by many parameters, such as fiber types, fiber distribution, or orientation, and can be commonly grouped in two distinct classifications: strain softening and strain hardening [13]. Compared with strain-softening ones, a strain-hardening material is considered to be more excellent with respect to mechanical performance. Nevertheless, due to the obvious distinction between the behavior of bending and uniaxial tension, strain-softening behavior in uniaxial tension can result in a deflection hardening response in bending, as shown in Figure 1. Due to bridge effect of fiber, the load–deflection response of UHPFRC prisms is significantly different with non-fiber-reinforced concrete, which always tends to brittle bending failure. Accordingly, it is still a challenging matter for researchers to determine the tensile properties of UHPFRC, resulting in possible vacillations among designers, inspectors, and administrators for counting on the participation of tensile performances in UHPFRC.

In order to specifically quantify the tensile behavior of FRCC, several standard test methods have been established, which can be classified into direct and indirect tests [14,15]. Due to the tensile property obtained directly without relying on inverse analysis, the direct test is confirmed to be the most suitable method to characterize the tensile property of UHPFRC with strain-hardening behavior. However, performing direct tests is time-consuming, challenging, and error-prone, especially for testing UHPFRC with strain-softening behavior, which is significantly influenced by many factors, including specimen size, boundary constraint, loading machine stiffness, eccentric tension, and the inhomogeneity of the material itself. Although uniaxial tensile tests that are easy to conduct have been developed by some researchers [16,17,18,19], it is not appropriate for quality control on construction sites.

As a simpler alternative to the direct tensile test, bending tests, such as the notched three-point bending test (3PBT) according to NF P18-470 [15] and EN-14651 [20], the unnotched four-point bending test (4PBT) according to ASTM C1609/C1609M-12 [21] and JCI-S-003-2007 [22], or the notched 4PBT according to CNR-DT 204 [23], are preferred in (HP)FRCC testing. The 3PBT with notched prisms is normally conducted to characterize the postcracking tensile law and to test UHPFRC materials that exhibit limited strain-hardening or strain-softening behavior, while the 4PBT is adopted to determine elastic properties and to test UHPFRC with significant strain-hardening behavior. As a result of the easier handling, bending tests are widely performed to characterize the tensile behavior of UHPFRC and its control quality in the field. However, we cannot regard the result obtained from bending tests as the intrinsic material’s tensile properties, and so further description and sophisticated inverse analysis are required before these properties can be applied to structural design.

Several inverse analysis methods have been developed for determining the tensile properties of FRCC based on bending tests [24]. All inverse methods are committed to converting the mechanical responses, such as load–deflection, load–crack mouth opening displacement (CMOD), and load–curvature, obtained from bending tests into uniaxial tensile properties by virtue of more or less complicated models that relate closely to constitutive parameters in uniaxial tension and compression [25,26,27]. These methods can be classified in two distinct categories: simplified methods and accurate methods. Simplified methods, which are based on the equilibrium of moments and forces in a sectional analysis, utilize only a few key points from the data obtained from the bending tests to derive several specific point coordinates on the stress–strain curve [28,29]. Simplified methods are suitable for structural design or quality control, as it is easy to implement. Nevertheless, these simplified methods based on the presumed constitutive model are inaccurate enough to generate a point-by-point curve [30,31]. On the basis of a complete experimental law, accurate methods utilize the load–deflection, load–CMOD, or load–curvature response to fit the uniaxial constitutive parameters and to get a point-by-point curve [24]. Due to a higher accuracy and sophisticated procedure, accurate methods are used for academic study purposes and are indicated for computer analyses.

However, it should be noted that both the simplified and accurate methods proposed in the most studies are based on 4PBTs, whereas the research about the inverse method for predicting the postcracking tensile behavior of strain-hardening and strain-softening UHPFRC based on load–deflection response that is obtained from the notched 3PBT is limited. Additionally, in order to make the notched 3PBT widely accepted for the structural design and quality control of UHPFRC, further simplification and validation are indispensable. The inverse analysis can be quick, easy, and reliable by developing a more approximate formulation that associates the midspan deflection with the curvature, and a united closed form solution for calculating the moment–curvature relationship of the UHPFRC specimen [32]. Furthermore, the reliable tensile information, which is used for designing UHPFRC elements, can be obtained by applying such a powerful inverse analysis method to the notched 3PBT.

Therefore, in order to efficiently obtain more realistic results, a nonlinear inverse analysis method based on the notched 3PBT was proposed in this paper. To evaluate the sensitivity of the predicted tensile behavior of UHPFRC to variations in the fiber volume fraction, a total of fifteen notched UHPFRC prisms with coarse aggregates and different contents of fiber were fabricated and tested in the 3PBT. A segmented tensile constitutive law was developed which is capable of describing the various tensile behaviors of UHPFRC, such as strain softening and strain hardening. Considering the effects of notch and shear force, a more approximate formulation was adopted to simulate the load–deflection response of the UHPFRC beam specimens. The analytical solutions were derived with a controllable accuracy that can be applied to different purposes, including academic study, structural design, or quality control, and verified with the direct tensile test results and existing inverse methods presented in Refs. [12,33]. A predictive application was developed which can be easily implemented with Matlab or Excel. Parametric studies were also performed for investigating the robustness of the proposed method.

## 2. Experimental Program

### 2.1. Materials and Test Setup

The mix proportions of the UHPFRC matrix used in this study are listed in Table 1. Basalt aggregate with the maximum size of 8 mm was included as a coarse aggregate. A type of brass-coated straight steel fiber with a diameter of 0.2 mm and with a length of 16 mm was used. The properties of the smooth steel fiber adopted in this study are shown in Table 2. To estimate the effect of the steel fiber volume content on the flexural performance, the experimental program included five groups with different fiber volume fractions of 0.5%, 0.75%, 1.0%, 1.25%, and 1.5%.

**Figure 2:**
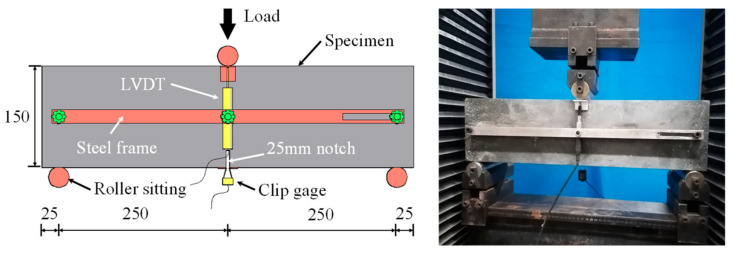
Notched three-point bending test (unit: mm).

A total of fifteen UHPFRC prisms (three prisms for each group) with dimensions of 150mm × 150mm × 550mm were fabricated for the notched 3PBT in accordance with EN 14651 [20]. In order to obtain the compressive performance, three cylinders for each group with a diameter of 100 mm and a length of 200 mm were also made. Additionally, all the prismatic specimens and cylindrical specimens were covered with plastic sheets and stored at room temperature for 24 h after casting, and then cured in water at a temperature of 20 °C for 28 days. The uniaxial compressive strengths of the UHPFRC were obtained by using a hydraulic compression testing machine with a maximum load capacity of 3000 kN at a rate of 0.1 mm/min. The three-point bending tests were conducted by using a universal testing machine with an ultimate load capacity of 300 kN at a constant rate of 0.2 mm/min. In order to initiate the crack location, a notch with a width of 5 mm and with a depth of 25 mm was sawn in middle of the lower side of the UHPFRC prism. The clear span is 500 mm. To measure the midspan deflection, two linear variable displacement transducers (LVDTs) were installed on both sides of the prism by using a steel frame. Additionally, a clip gage with a rated capacity of 8 mm was located at the notch for the CMOD measurement. The test setup and geometry for the 3PBT with notched prism are illustrated in Figure 2.

### 2.2. Experimental Results

For the UHPFRC-notched prism subjected to a center point load, the bending stress can be calculated by using Equation (1) according to EN 14651 [20].

(1) $$f=\frac{3PL}{2b{\left(d-a\right)}^{2}}$$

where *f* and *P* represent the bending stress and applied load, respectively; *a*, *b*, *d*, and *L* denote the notch depth, beam width, beam depth, and clear span, respectively.

**Figure 3:**
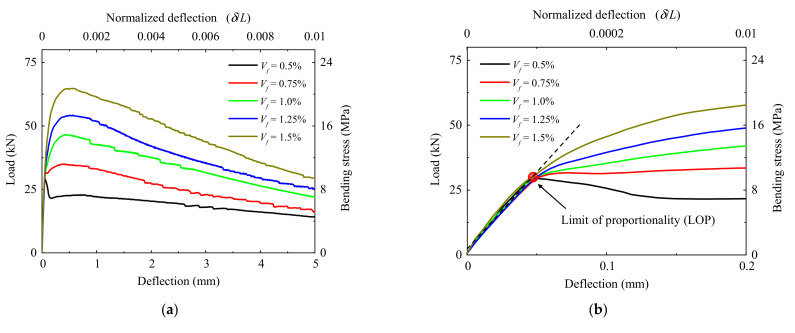
Load–deflection responses of UHPFRC prisms with various fiber contents: (**a**) The overall of load–deflection responses; (**b**) Initial part of load–deflection responses.

**Figure 4:**
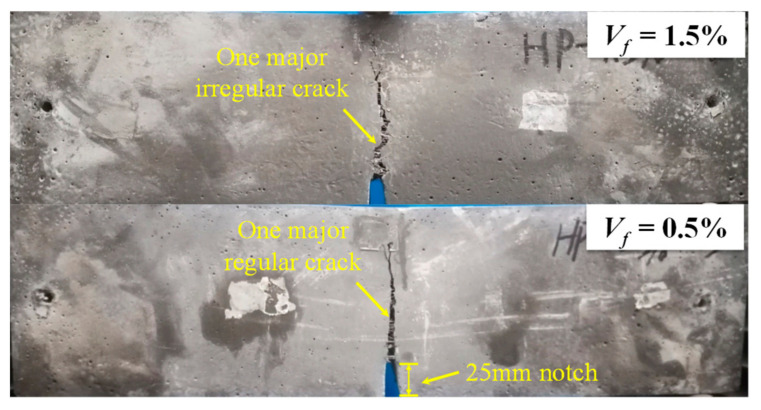
Crack patterns of beam specimens with fiber volume content of 0.5% and 1.5%.

The load–deflection curves obtained from the notched 3PBT for all test groups are shown in Figure 3. The averaged load–deflection responses indicated by bold lines were obtained from three specimens for each group. The limit of proportionality (LOP) in the load–deflection curve can be considered as the first cracking point of the UHPFRC prisms with smooth steel fibers, according to the study of Yoo et al. [33], as shown in Figure 3. The initial stiffness, the first cracking load, and the corresponding deflection at LOP were not sensitive to variations in the fiber volume content, whereas the peak load and the descending slope were significantly affected by the fiber volume fraction. In addition, the deflection at the point of modulus of rupture (MOR) corresponding to peak load exhibited no noticeable difference for the specimens with the fiber volume fractions higher than 0.5%. For the beam with a fiber volume fraction of 0.5%, a sudden increase of deflection with a sudden load drop was observed after matrix cracking, whereas the deflection-hardening behavior was obtained for the beams with the fiber volume fraction higher than 0.5%. It should be noted that only one major crack was observed at the midspan of all specimens, as shown in Figure 4. As a higher volume content of fiber was adopted, more irregularity of the crack was exhibited along the beam depth. Additionally, the characteristic parameters with average value for depicting the UHPFRC compressive performance and bending behavior are summarized in Table 3.

## 3. Derivation of Nonlinear Inverse Method

### 3.1. Proposed Stress–Strain Relationship

**Figure 5:**
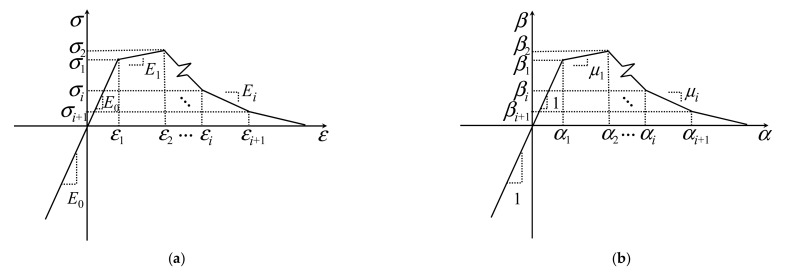
Material model of UHPFRC: (**a**) The stress–strain law; (**b**) The normalized constitutive law.

The nonlinear inverse method entails having to assume a roughly stress–strain relationship based on several assumptions in both tension and compression. With high compressive strength, UHPFRC is typically considered to keep up higher linearity before the maximum load [33]. Therefore, the compressive constitutive law of UHPFRC is supposed to be linear–elastic at any stress, as shown in Figure 5a. Although this assumption may cause error at a high level of compressive strain, the formulation can be developed without requiring any compressive parameter [32]. In addition, the uniaxial tensile stress–strain relationship of the UHPFRC is typically nonlinear, but any stress–strain curve envelop can be represented by a series of piecewise linear functions. As shown in Figure 5a, a constitutive model in tension is proposed with a segmented function that is able to characterize any experimental curve obtained from uniaxial tensile tests. The elastic modulus (*E*_0_) for the UHPFRC is considered the same both in compression and tension. In Figure 5a, *E_i_* represents the modulus in the (*I* + 1)th region of the tensile response, such as *E*_1_ represents the postcracking modulus that can simulate either strain-hardening or softening materials by being assigned a positive or negative scalar value, respectively; *σ_i_* and *ε_i_* represent the stress and the corresponding strain at a point where the slope of the tensile curve changes, such as *σ*_1_ and *ε*_1_ represent the tensile strength and strain at LOP, respectively. The mathematical form of the tensile and compressive stress–strain relationship is depicted as follows:

(2) $$\sigma (\varepsilon )=\left\{\begin{array}{l} E_{0}\varepsilon \;\;\;\;\;\;\;\;\;\;\;\;\;\;\;\;\;\;\;\;\;\;\;\;\;\;\;for\;(-\infty \leq \varepsilon <\varepsilon_{0})\\ \sigma_{i}+E_{i}(\varepsilon -\varepsilon_{i})\;\;\;\;\;\;\;for\;(\varepsilon_{i}\leq \varepsilon <\varepsilon_{i+1},i=0,\ldots ,n) \end{array}\right.$$

The formulation can be further simplified by introducing three normalized parameters: *α_i_*, *β_i_* and *μ_i_*, as defined in Equation (3). The normalized stress–strain diagram is described in Figure 5b.

(3) $$\alpha_{i}=\frac{\varepsilon_{i}}{\varepsilon_{1}};\;\beta_{i}=\frac{\sigma_{i}}{\sigma_{1}};\;\mu_{i}=\frac{E_{i}}{E_{0}}=\frac{\beta_{i+1}-\beta_{i}}{\alpha_{i+1}-\alpha_{i}}\left(i=0,\ldots ,n\right)$$

The following normalized stress–strain relationship is developed by substituting all normalized parameters expressed in Equation (3) into Equation (2).

(4) $$\beta =\left\{\begin{array}{l} \alpha \;\;\;\;\;\;\;\;\;\;\;\;\;\;\;\;\;\;\;\;\;\;\;\;\;\;\;\;\;\;\;for\;(-\infty \leq \alpha <\alpha_{0})\\ \beta_{i}+\mu_{i}(\alpha -\alpha_{i})\;\;\;\;\;\;\;for\;(\alpha_{i}\leq \alpha <\alpha_{i+1},i=0,\ldots ,n) \end{array}\right.$$

### 3.2. Derivation of Closed Form Moment–Curvature Formulation

**Figure 6:**
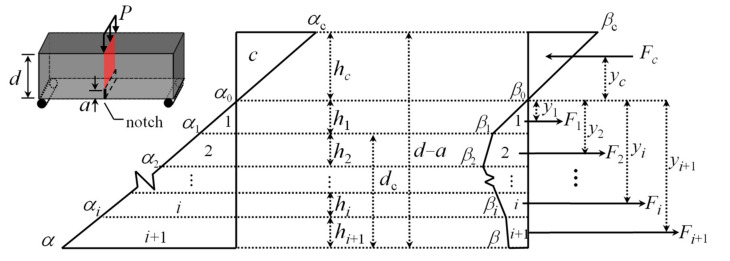
Linear distribution of strain along the depth and the corresponding stress distribution.

Considering that a rectangular section with a depth (*d* – *a*) and width (*b*) above the notch is shown in Figure 6, three assumptions are introduced in the derivation of the moment–curvature (*M*−*ϕ*) formulation: (1) resultants of internal stress equilibrate with the externally applied loads; (2) plane sections remain plane during bending; (3) the stress–strain relationship of the material is assumed.

Assuming that strain distributes linearly along the depth of notched cross-section, as shown in Figure 6, the stress distribution across the section can be obtained depending on the proposed stress–strain relationship in Figure 6. Both the normalized strain at the top fiber (α_c_) and the normalized strain at the bottom fiber (α) are linearly related to the neutral axis depth ratio (*k*), as presented in Equation (5). The normalized height and the internal force of each component obtained from the stress–strain diagram are presented in Equations (6) and (7), respectively.

(5) $$\alpha_{c}=\frac{k}{1-k}\alpha$$

(6) $$h_{c}=k;\;h_{j}=\frac{\left(\alpha_{j}-\alpha_{j-1}\right)}{\alpha }(1-k)\;\left(j=1,\ldots ,i\right);\;h_{i+1}=\frac{\left(\alpha -\alpha_{i}\right)}{\alpha }(1-k)$$

(7) $$F_{c}=\frac{\alpha k^{2}}{2(1-k)};\;F_{j}=\frac{\left(\alpha_{j}-\alpha_{j-1})(\beta_{j}+\beta_{j-1}\right)}{2\alpha }(1-k);\;F_{i+1}=\frac{\left(\alpha -\alpha_{i})(\beta +\beta_{i}\right)}{2\alpha }(1-k)$$

Similarly, the normalized moment arms, which are measured from the centroid of each force component to the neutral axis, are presented in Equation (8).

(8) $$y_{c}=\frac{2}{3}k;\;y_{j}=\frac{C_{j}(1-k)}{3\alpha (\beta_{j}+\beta_{j-1})};\;y_{i+1}=\frac{C_{i+1}(1-k)}{3\alpha (\beta +\beta_{i})}$$

where *C_j_* = 2*α_j_β_j_* + *α_j_β_j_*_−1_ + *α_j_*_−1_*β_j_* + 2*α_j_*_−1_*β_j_*_−1_ (*j* = 1, ···, *i*); *C_i+_*_1_ = 2*αβ* + *α_i_β*+ *αβ_i_* + 2*α_i_β_i_* (*I* = 1, ···, *n*).

The neutral axis depth ratio (*k*) is presented in Equation (9), which can be solved by the equilibrium of internal forces. Additionally, the internal moment (*M*) is determined as a summation of each force component multiplied by their respective moment arms. Likewise, the curvature (*ϕ*) is considered as the ratio of the compressive strain at the top fiber to the depth of the neutral axis. *M* and *ϕ* are normalized with respect to their respective values at LOP, and the normalized forms *M*’ and *ϕ*’ are presented in Equations (10) and (11), respectively.

(9) $$k=\frac{D_{i}}{\alpha +D_{i}};\;D_{i}=\sqrt{(\beta +\beta_{i})(\alpha -\alpha_{i})+\sum_{j=1}^{i}\left(\beta_{j}+\beta_{j-1})(\alpha_{j}-\alpha_{j-1}\right)}\;\left(i=0,\ldots ,n\right)$$

(10) $$M^{\prime }=\frac{2\alpha k^{3}}{1-k}+\frac{{\left(1-k\right)}^{2}}{\alpha^{2}}\left[C_{i+1}(\alpha -\alpha_{i})+\sum_{j=1}^{i}C_{j}(\alpha_{j}-\alpha_{j-1})\right]$$

(11) $$\phi^{\prime }=\frac{\alpha }{2(1-k)}$$

(12) $$M=M^{\prime }M_{LOP};\;M_{LOP}=\frac{1}{6}b{\left(d-a\right)}^{2}E_{0}\varepsilon_{1}$$

(13) $$\phi =\phi^{\prime }\phi_{LOP};\;\phi_{LOP}=\frac{2\varepsilon_{1}}{d-a}$$

When the number of segments (*n*) is equal to 3, the same moment–curvature (*M*−*ϕ*) formulation derived by Soranakom and Mobasher [30] can be obtained from Equations (10) and (11).

In a sectional analysis, the *M*−*ϕ* relationship of the UHPFRC beams can be obtained by three procedures. Firstly, an initial value is selected for the tensile strain at the bottom fiber (α), then the maximum compressive strain (α_c_) and the neutral axis depth ratio (*k*) are calculated by Equations (5) and (9), respectively. Secondly, the internal moment (*M*) and corresponding curvature (*ϕ*) can be determined using Equations (12) and (13), respectively. In this way, one point is produced on the *M*−*ϕ* diagram. At last, to describe the complete moment–curvature response, sufficient points should be generated by repeating the first and second steps.

### 3.3. Load–Deflection Response

**Figure 7:**
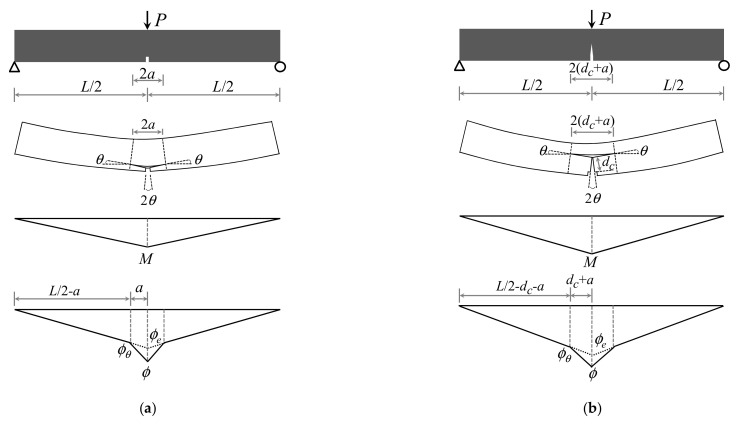
Moment and curvature distributions along the notched beam at two stages: (**a**) Precracking; (**b**) Postcracking.

Although a closed form moment–curvature formulation was derived, the relationship should be developed to connect the curvature and the midspan deflection. As for a notched beam submitted to three-point bending, the elastic behavior of the region near the notch is typically perturbed before cracking, as the stress field is modified by the notch. According to previous research [34], the length of the perturbed area is roughly twice the notch depth. As shown in Figure 7a, the length of the perturbed area is assumed to be 2*a* at the elastic stage in this study. As for a notched beam loaded beyond the cracking strength, a single macrocrack is usually observed above the notch [33]. Hence, the region near the notch is also perturbed by the crack depth (*d*_c_), and the length of this region is changed to be 2(*d*_c_ + *a*), as shown in Figure 7b. In this region, beam theory is applied to the analysis of an uncracked part above the macrocrack, and two rigid blocks at the bottom are considered to behave in accordance with the kinematic hypothesis. The consistency of the rotation angle is satisfied at the boundaries of the perturbed zone.

**Figure 8:**
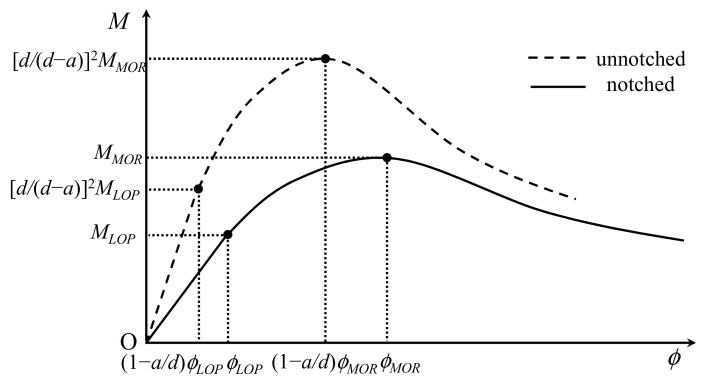
Moment–curvature relationships in notched and unnotched cross section.

Due to the influence of the notch, the moment–curvature relationship of the section at midspan, which is described by dashed curve in Figure 8, is different with that of the section at the boundaries of the perturbed area described by the solid curve. To simplify the analysis, a linear variation of curvature is assumed from the value *ϕ* in the midspan to the value *ϕ_θ_* in a length (*d*_c_ + *a*), as shown in Figure 7b. When the moment in the midspan beyond ultimate moment at MOR (*M_MOR_*), the areas near the crack follow the softening portion of solid curve in Figure 8, whereas the remainder of beam specimen undergoes unloading elastically. Moreover, *ϕ_θ_* is denoted in Equation (14) with respect to the midspan curvature (*ϕ_LOP_*). The crack depth (*d*_c_) above the notch and the rotation angle (*θ*) at the boundaries of the perturbed zone, shown in Figure 7b, are presented in Equations (15) and (16), respectively.

(14) $$\phi_{\theta }=[1-\frac{2(d_{c}+a)}{L}]{\left(1-\frac{a}{d}\right)}^{3}\frac{M}{M_{LOP}}\phi_{LOP}$$

(15) $$d_{c}=\frac{\left(\alpha -1)(1-k)(d-a\right)}{\alpha }$$

(16) $$\theta =\int_{0}^{d_{c}+a}\phi (x)dx=\frac{\left(\phi +\phi_{\theta })(d_{c}+a\right)}{2}$$

where *M_LOP_* and *ϕ_LOP_* are the moment and curvature of the central section at LOP, respectively, and *a* and *d*_c_ are the depth of notch and crack, respectively.

As described in Equation (17), the total midspan deformation consists of three components, including the deflection (*δ_m_*) caused by the bending deformation in the elastic state, the deflection (*δ_v_*) caused by the shear deformation, and the deflection (*δ_c_*) caused by crack propagation above the notch. Compared with the bending deformation, the shear distortion is generally ignored under the large span-to-depth ratio. Conversely, the deflection caused by shear distortion cannot be neglected due to the small span-to-depth ratio in this study. The shear deflection (*δ_v_*) can be computed according to the shear strain distribution along the specimen [31]. Furthermore, the relationship between the shear force and strain is supposed to be linear–elastic if no shear cracks are observed.

(17) $$\delta =\delta_{m}+\delta_{v}+\delta_{c}=\int_{-L/2}^{L/2}(M_{u}\phi_{e})dx+\int_{-L/2}^{L/2}(V_{u}\gamma )dx+\frac{\theta L}{2}$$

where *ϕ_e_* is the curvature of a beam in the elastic state, as shown in Figure 7; *γ* is shear strain along the notched specimen; *M_u_* and *V_u_* represent the unit virtual moment and the unit virtual shear force, respectively.

By applying diagram multiplication to both the shear force–strain and moment–curvature diagrams, the midspan deflection formula of the notched beam can be derived explicitly as follows:

(18) $$\delta ={\left(1-\frac{a}{d}\right)}^{3}\frac{M\phi_{LOP}}{12M_{LOP}}\left[L^{2}+2\kappa (1+\nu )d^{2}\right]+\frac{\theta L}{2}$$

where *κ* is the shear form factor, which is equal to 1.2 for rectangular cross-section, and *ν* is Poisson’s ratio. The applied load *P* can be calculated as follows:

(19) P=4M/L

Then, the load–deflection response can be obtained by a combination of Equations (18) and (19).

### 3.4. Algorithm to Predict the Tensile Properties

**Figure 9:**
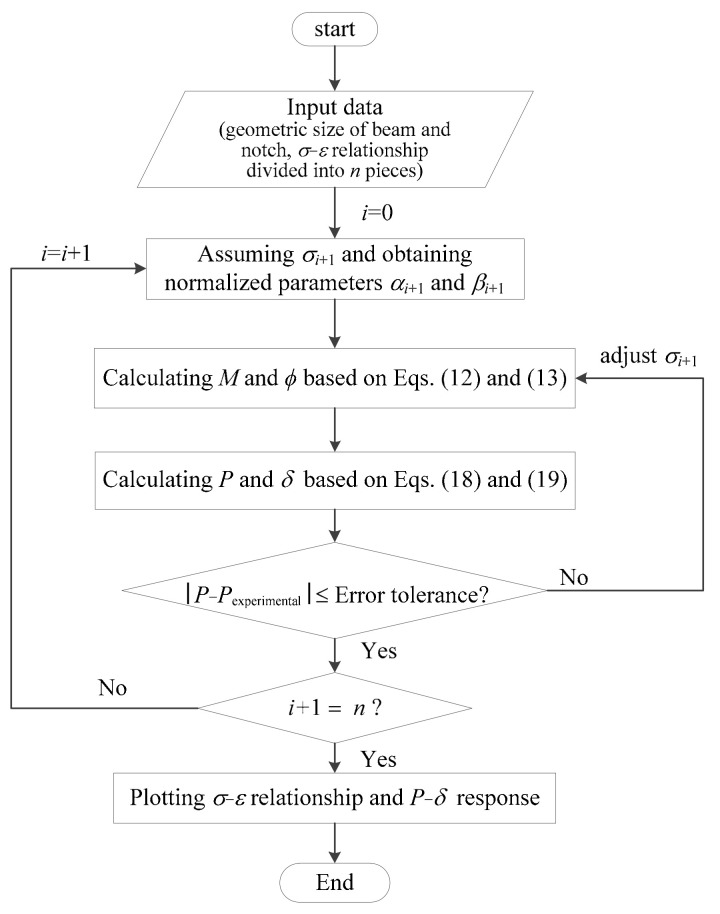
Implementation procedure for the nonlinear inverse analysis.

In the inverse analysis, both the load–deflection and the moment–curvature responses were developed on the basis of a stress–strain relationship proposed in this study. The proposed inverse analysis method can be conveniently implemented in Matlab or Excel with the analytical solutions derived above. The implementation procedure is shown in Figure 9. The following procedures are appropriate for designers to obtain the tensile properties based on the notched 3PBT.

1. Firstly, the *σ*-*ε* relationship is divided into *n* pieces according to the different precision requirement. Then, some initial parameters, including specimen size and constant coefficient, should be determined;
2. An initial value is assigned to the stress *σ*_i+1_, and then the moment–curvature response can be generated using Equations (12) and (13). Next, the load–deflection response for a notched beam can be calculated by using Equations (18) and (19);
3. The stress *σ*_i+1_ is adjusted, and then step 2 is repeated until the calculated load–deflection response fit the experimental results within acceptable error tolerance;
4. The stress–strain relationship and the approximate load–deflection curve can be obtained by repeating steps 2 and 3.

## 4. Verification of Nonlinear Inverse Method

As the localization of the distortions takes place at the macrocrack in the direct tensile tests, the stress–strain relationship is no longer appropriate for the description of the softening behavior after crack localization. In general, the softening behavior of the UHPFRC in the uniaxial tensile tests is described by stress–crack opening (*σ*-*w*) curves. Nevertheless, the tensile properties of the UHPFRC obtained by the proposed inverse analysis are described by the stress–strain (*σ*-*ε*) law. In order to obtain an equivalent strain, a reference length (*l*_cs_), expressed in Equation (20), is introduced to convert the *σ*-*w* relationship into the *σ*-*ε* diagram. The reference length is assumed as the beam depth (*d*) for simplicity, according to fib Model Code 2010 [35].

(20) $$\varepsilon =\sigma (w)/E_{0}+w/l_{cs}$$

### 4.1. Comparison with Direct Tensile Test Results

The experimental results obtained by Leutbecher and Rebling [12] were used for checking the results obtained from the nonlinear inverse method. In their experimental program, they fabricated and tested a number of UHPFRC prisms (150 mm × 150 mm × 550 mm) with a 25 mm notch at midspan. In order to minimize the influence of the fiber orientation and distribution, strip-shaped members with a length of 400 mm were cut from the UHPFRC beam specimens and tested in uniaxial tensile tests. A total of six series with various parameters, such as maximum aggregate size, fiber geometry, and fiber volume fraction, were included in their test program, whereas only specimens of series 1 and series 2 were selected and discussed here, as no stress–crack opening curve was available for series 3–6.

In the inverse analysis, the average load–deflection curve is used as the target curve. Moreover, the average stress-crack opening curve obtained from the direct tensile test is converted into a stress–strain curve depending on a reference length of 150 mm. The mean cube strengths for series 1 and series 2 are determined to be 176 and 172 MPa, respectively. The details of the two series and the essential parameters used in the inverse analysis are listed in Table 4.

**Figure 10:**
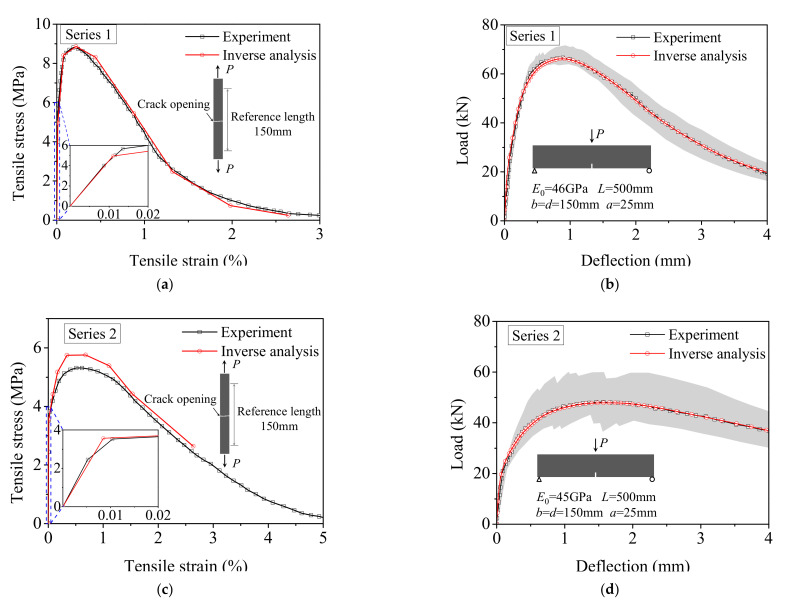
Comparison of inverse analysis results with experimental results reported in Ref. [12]: (**a**) Tensile stress–strain curves for series 1; (**b**) Load–deflection responses for series 1; (**c**) Tensile stress–strain curves for series 2; (**d**) Load–deflection responses for series 2.

Figure 10 depicts the comparison of the tensile *σ*-*ε* curve back-calculated by the proposed method with the uniaxial tensile test results and the correlation between the calculated and measured load–deflection responses for series 1 and series 2. Figure 10a shows a *σ*-*ε* curve calculated by the analysis method is in good agreement with the direct tensile test results of series 1. However, the predicted tensile strength is slightly higher than the test result for series 2 due to the underestimation of elastic modulus, as shown in the inset of Figure 10c, and the higher scatter of experimental load–deflection responses, as illustrated in Figure 10d. In addition, since no experimental data are available for the deflection larger than 4mm, the stress–strain curve calculated by the inverse analysis is incomplete for series 2. The maximum compressive strain obtained by using Equation (5) is 2.01 × 10^−3^, which is only half of the ultimate compressive strain. This result indicates that the compressive constitutive law assumed to be linear–elastic at any stress is applicable for the inverse analysis of UHPFRC.

### 4.2. Comparison with Existing Inverse Method

In order to validate the proposed inverse method, the experimental results reported in the study of Yoo et al. [33] were used. In their three-point bending tests, the UHPFRC beam specimens (100 mm × 100 mm × 400 mm) with a 10 mm notch at the midspan were casted and tested. The sets of flexural test specimens and the UHPFRC mechanical properties are listed in Table 5. Additionally, Yoo et al. [33] suggested a bilinear softening curve for the UHPFRC based on the result of inverse analysis, which could be used to check the results obtained from the proposed analysis method. In their inverse analysis, the finite element model with dense meshing was used.

**Figure 11:**
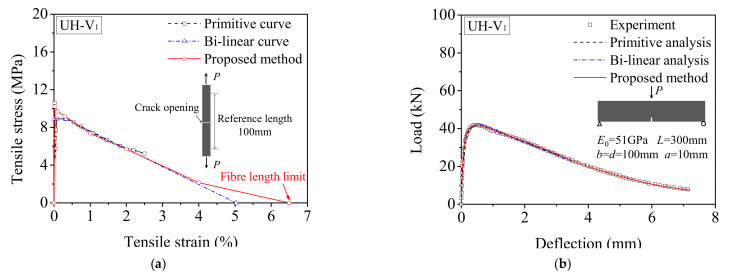

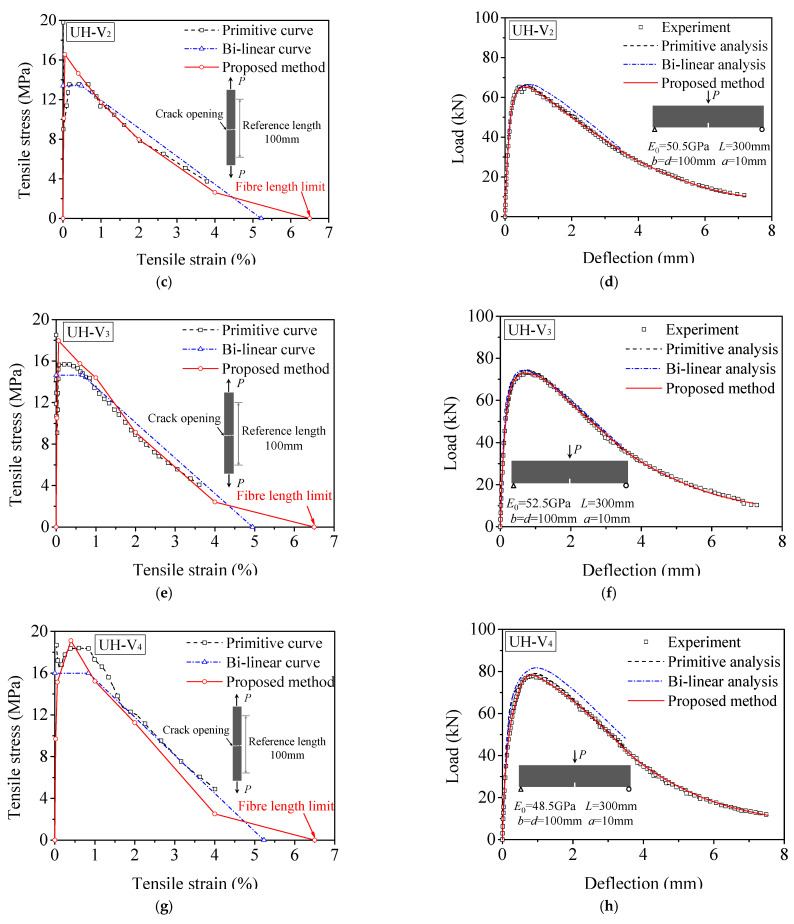
Comparison of inverse analysis results with primitive and bilinear softening curves for each group reported in Ref. [33]: (**a**) *σ*-*ε* curves for UH-V_1_; (**b**) *L*-*δ* responses for UH-V_1_; (**c**) *σ*-*ε* curves for UH-V_2_; (**d**) *L*-*δ* responses for UH-V_2_; (**e**) *σ*-*ε* curves for UH-V_3_; (**f**) *L*-*δ* responses for UH-V_3_; (**g**) *σ*-*ε* curves for UH-V_4_; (**h**) *L*-*δ* responses for UH-V_4_.

Similarly, both the primitive and bilinear softening curves suggested by Yoo et al. [33] were converted into the tensile *σ*-*ε* curve depending on a reference length of 100 mm. The comparisons of the predicted tensile *σ*-*ε* curves obtained from the proposed inverse analysis with the analysis of Yoo et al. [33] are presented in Figure 11. The maximum tensile strain 6.5% corresponding to the cracking opening of 6.5 mm in accordance with the fiber length limit is obtained from the proposed analysis method. Compared with the primitive analysis, the results derived from the bilinear analysis seem to slightly overestimate the experimental load–deflection in the descending branch, especially for UH-V_2_ and UH-V_4_. The stress–strain response obtained from the proposed method provides the best fit of the primitive softening curve for UH-V_1_, UH-V_2_, and UH-V_3_. A slight deviation between the proposed analysis and the primitive analysis result for UH-V_4_, as shown in Figure 11g, is mainly due to the intrinsic scatter of the experimental load–deflection responses at a high strength level and the low disperse degree of the fiber. The first cracking tensile strength with an approximate value of 10MPa is obtained by the proposed method for all specimens. This means that, due to effect of the fiber volume fraction, UH-V_1_ exhibits a strain-softening behavior while UH-V_2_, UH-V_3_, and UH-V_4_ exhibit a strain-hardening behavior. Therefore, the verification results indicate that the proposed method can be used for predicting the postcracking tensile behavior of UHPFRC.

### 4.3. Summary of Method Verification

In summary, the method verification indicated that the proposed inverse method could reasonably predict the UHPFRC tensile properties, including the softening and hardening parameters, based on the load–deflection response that was obtained from the notched 3PBT. The verifying results indicated the correlation between the measured and back-calculated tensile σ-ε responses was improved. Actually, the underestimation of the first cracking tensile strain and the elastic modulus could lead to the overestimation of the ultimate tensile strength. Therefore, it is essential to well calibrate the initial parameters, including the first cracking tensile strain and elastic modulus. Meanwhile, the analytical result of the proposed method was significantly affected by the scatter of the experimental load–deflection responses.

## 5. Application of the Proposed Method for Parametric Studies

In order to estimate the effect of the fiber volume fraction on the tensile performance of UHPFRC, the proposed method was adopted for predicting the tensile property based on the bending test results shown in Section 2. Additionally, parametric studies were also conducted for evaluating the robustness of the proposed method.

### 5.1. Application of Predicting Tensile Behavior

**Figure 12:**
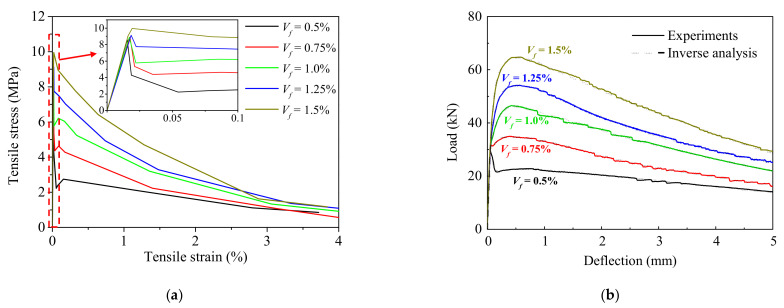
The sensitivity of the predicted tensile behavior to variations in fiber volume fraction: (**a**) The predicted tensile stress–strain curves; (**b**) The correlations between calculated and experimental load–deflection responses.

The bending test results shown in Figure 3 and the parameters listed in Table 1 were used for the inverse analysis. The tensile stress–strain curves of the UHPFRC with different fiber volume fractions were well predicted by using the proposed method to fit the load–deflection responses, as shown in Figure 12a. Additionally, the correlations between calculated load–deflection response and that obtained from the notched 3PBT were depicted in Figure 12b. As shown in inset of Figure 12a, the postcracking tensile strength exhibits an approximately proportional behavior to the fiber volume fraction, whereas the first cracking tensile strength was insensitive to the fiber volume fraction, and it was primarily determined by the matrix strength. In addition, the ultimate tensile strain was also seldom affected by the amount of fiber, which is mainly influenced by the fiber length and orientation. It should be noted that a strain-softening behavior was observed for the UHPFRC with the fiber volume fraction lower than 1.5%, while a limited strain-hardening behavior was obtained for the UHPFRC with the fiber volume fraction of 1.5%. For a strain-softening UHPFRC with deflection-hardening behavior in bending, the deflection corresponding to the bending strength was insensitive to the fiber volume fraction.

As shown in Figure 12a, due to the fiber bridging, the strain-softening part of tensile stress–strain response contributes to the load-carrying capacity and nonlinear energy dissipation. When subjected to bending stresses, the post-peak response in the tensile regions contributes to the load-carrying capacity in the softening observed in the deflection response of the UHPFRC prisms with the fiber content of 0.5%. However, if the volume fraction of the fibers is larger than 0.75%, as shown in Figure 12b, the stiffness contribution of the cracked zone may result in loads in excess of the first cracking point and is defined as deflection hardening. Therefore, the stiffness of the cracked zone in the tensile regions contributes to the increased capacity in bending at large deflection levels.

### 5.2. Effect of the Number of Segments

In the nonlinear inverse method, the tensile constitutive law is divided into n pieces, as defined in Equation (2). In general, the larger the number of segments (*n*) is, the more accurate the calculation result. Nonetheless, the implementation of the proposed method is more complex with increasing the number of segments. Hence, it is essential to make a balance between the handiness and the accuracy of the proposed method.

**Figure 13:**
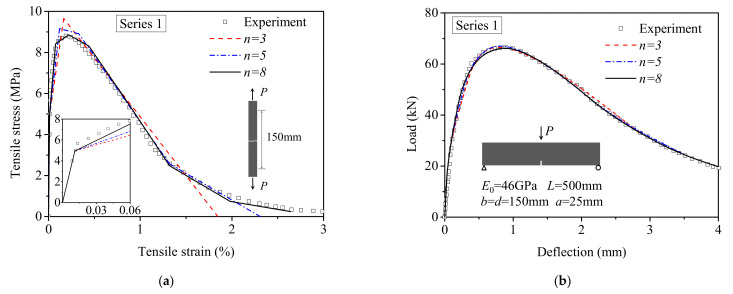
Effect of the number of segments (*n*) on the accuracy of proposed method: (**a**) The predicted tensile stress–strain curves; (**b**) The calculated and experimental load–deflection responses.

Figure 13 shows the effect of the number of segments on the accuracy of the proposed inverse method. The overestimation of the calculated postcracking strength is reduced from 9.6% to 0.6% compared to experimental result, with the number of segments increased from three to eight. Furthermore, the modulus of the strain-hardening stage is improved with increasing the number of segments, as shown in the inset of Figure 13a. When *n* is equal to 5, the corresponding overestimation is under 4%, which is precise enough for the structural design and quality control of the strain-hardening UHPFRC. It shows that a highly precise analytical solution that agrees with the results of the direct tensile test can be obtained as long as the length of each segment is small enough. Therefore, the proposed method can be also used for research purposes.

### 5.3. Effect of Notch-to-Depth Ratio

With respect to the notched 3PBT, both the moment and curvature at the midspan are influenced by the notch-to-depth ratio (*a*/*d*), as shown in Figure 9. It is observed that the curvature at midspan increases continuously, and the moment decreases rapidly with an increasing notch-to-depth ratio. Meanwhile, the curvature distribution along the length of the perturbed area is also affected by the notch-to-depth ratio and further influences the load–deflection response. In order to investigate the influence of the notch-to-depth ratio, the load–deflection responses are simulated for series 1 with a notch depth of 0, 12.5, 25, and 50 mm at the midspan of the beam specimens that which result in a ratio *a*/*d* of 0, 0.08, 0.16, and 0.33, respectively.

**Figure 14:**
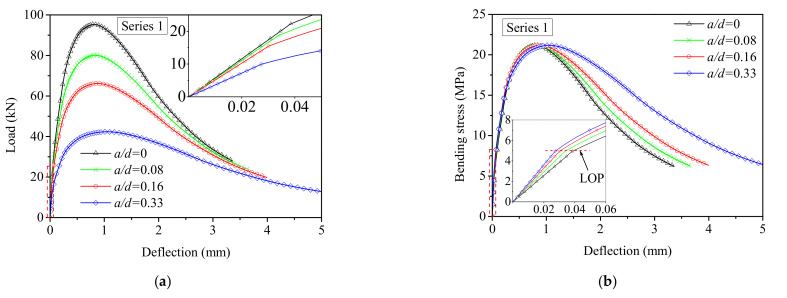
Effect of the notch-to-depth ratio (a/d) on: (**a**) Load–deflection responses; (**b**) Bending stress–deflection responses.

Figure 14a demonstrates the effect of the ratio *a/d* on the load–deflection response of series 1. It is observed that the peak load decreases sharply, and the curve becomes smooth with an increasing notch-to-depth ratio. As shown in the inset of Figure 14a, the load at LOP is inversely proportional to the notch-to-depth ratio because UHPFRC is more prone to cracking failure with the increase of that ratio. Furthermore, Figure 14b depicts the bending stress–deflection curves of series 1 with different notch-to-depth ratios. The bending stress was calculated by using Equation (1). The bending strength is slightly affected by the notch-to-depth ratio, and softening occurs more slowly with an increasing notch-to-depth ratio, as shown in Figure 14b. This is due to the distance between the top of the specimen and the tip of the notch, which reduces with the increase of the notch-to-depth ratio, leading to a lower first cracking load.

### 5.4. Effect of Postcracking Strength

**Figure 15:**
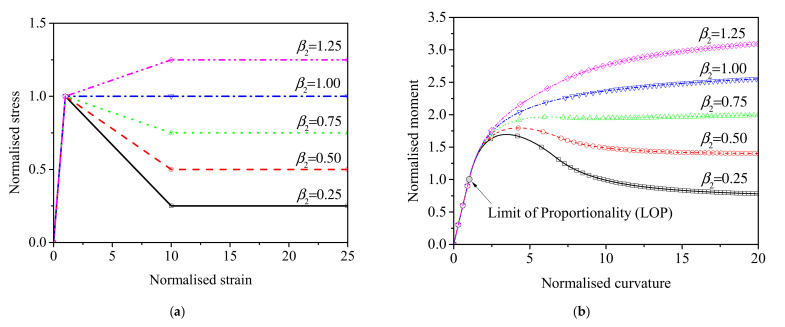
Effect of the normalized parameter *β*_2_ to: (**a**) The normalized tensile constitutive model; (**b**) The normalized moment–curvature response.

As for the postcracking strength study, the bending tensile strength and ductility were expressed as the normalized *M*−*ϕ* response, which is irrelevant to specimen size and first cracking tensile strength. Figure 15a demonstrates the tensile constitutive model with the normalized postcracking strength (*β*_2_) varied from 0.25 to 1.25 and the corresponding normalized transition strain *α*_2_ = 10, simulating a range of strain-softening response of the UHPFRC with a low fiber volume fraction, to the strain-hardening response of the UHPFRC with a high fiber volume fraction. Figure 15b illustrates that the normalized *M*−*ϕ* relationship is highly sensitive to variations in the material parameter *β*_2_, as it significantly influences the peak and post-peak response. It is important to remark that the bending tensile strength and ductility are improved as the normalized postcracking strength changes from 0.25 to 1.25, and a significant deflection hardening response occurs when *β*_2_ is larger than 0.75. This means that a strain-softening material can exhibit deflection-hardening or deflection-softening behavior in bending. Considering the significant deflection-hardening material is applicable for structural application where bending prevails, a postcracking strength no less than 75% of the first cracking strength is recommended for strain-softening UHPFRC.

## 6. Conclusions

In this paper, a nonlinear inverse method based on the notched 3PBT was developed for predicting the tensile properties of UHPFRC. An experimental program was conducted to evaluate the sensitivity of the predicted results. The verifications and parametric studies were also performed to investigate the generality and robustness of the proposed method. The main conclusions are as follows:

1. The verifications indicated that the segmented stress–strain model used in the nonlinear inverse analysis was capable of describing various tensile properties of the UHPFRC, including strain softening and strain hardening. Both the tensile strain–stress relationship and the load–deflection response showed good agreement between the experimental and analytical results. However, the accuracy of the predicted result was significantly affected by the scatter of the experimental load–deflection response;
2. A high sensitivity of the proposed method was observed with a fiber volume fraction varying from 0.5% to 4%. The deflection-softening behavior was simulated for the UHPFRC beams with the fiber volume fraction of 0.5%, in accord with test result. That the strain-softening behavior resulted in a deflection-hardening response was also identified by the prediction. For a strain-softening UHPFRC with deflection-hardening behavior in bending, the deflection corresponding to the bending strength was insensitive to the fiber volume fraction;
3. For the strain-hardening UHPFRC, the predicted ultimate tensile strength was highly sensitive to the number of segments. The overestimation of the ultimate tensile strength was less than 4% with increasing the number of segments to five. However, with reducing the number of segments to three, the overestimation of the postcracking strength reached around 10%. Therefore, this method with a controllable accuracy can be adapted for academic research and structural design;
4. The load–deflection response of the UHPFRC beam specimens was significantly affected by the notch-to-depth ratio. With a lower notch-to-depth ratio, the deflection-hardening behavior was more obvious, and softening occurred more quickly. Nonetheless, the bending strength was slightly affected by the notch-to-depth ratio;
5. For a strain-softening material, a significant deflection-hardening response was observed with the postcracking strength larger than 75% of the first cracking strength and the corresponding transition strain of 0.15%. Both the postcracking strength and the transition strain are most important factors to the bending strength and ductility of the strain-softening UHPFRC.

## Figures and Tables

**Table 1:** Mix proportions of UHPFRC material (proportion by weight).

Water	Cement	Silica Fume	Fine Aggregate	Coarse Aggregate	Superplasticizer
0.24	1	0.23	1.08	1.24	0.034

**Table 2:** Properties of straight steel fibers.

Diameter (mm)	Length (mm)	Aspect Ratio (*L_f_*/*D_f_*)	Elastic Modulus (GPa)	Tensile Strength (MPa)	Density (kg/m^3^)
0.2	16	80	200	2500	7850

Note: *L_f_* = fiber length, *D_f_* = fiber diameter.

**Table 3:** Summary of mechanical test results.

Test Group	*f_c_* (MPa)	*E*_0_ (GPa)	*P_MOR_* (kN)	*δ_MOR_* (mm)
*V_f_* = 0.5%	139.9(2.65)	55.40(0.68)	29.5(0.78)	0.05(0.001)
*V_f_* = 0.75%	135.2(3.21)	50.96(0.97)	35.2(0.64)	0.42(0.026)
*V_f_* = 1.0%	134.2(2.37)	52.24(1.39)	47.1(2.13)	0.47(0.065)
*V_f_* = 1.25%	140.6(1.80)	49.31(0.55)	54.4(0.35)	0.51(0.063)
*V_f_* = 1.5%	147.3(1.74)	56.52(0.83)	65.1(2.42)	0.53(0.059)

Note: *f_c_* = uniaxial compressive strength, *E*_0_ = elastic modulus, *P_MOR_* = peak load, *δ_MOR_* = deflection at the peak load, (x.xxx) = standard deviation.

**Table 4:** Details of UHPFRC mixtures and parameters used in inverse analysis.

Test Series	*L_f_*/*d_f_* (mm/mm)	*V_f_* (%)	*L* (mm)	*b* (mm)	*d* (mm)	*a* (mm)	*E*_0_ (GPa)	*κ*	*ν*
1	9/0.175	2.5	500	150	150	25	46.1	1.2	0.2
2	17/0.2	1	500	150	150	25	45.0	1.2	0.2

**Table 5:** Sets of flexural test specimens and UHPFRC mechanical properties.

Test Series	*L_f_*/*d_f_* (mm/mm)	*V_f_* (%)	*L* (mm)	*b* (mm)	*d* (mm)	*a* (mm)	*E*_0_ (GPa)	*f*_c_ (MPa)	*κ*	*ν*
UH-V_1_	13/0.2	1	300	100	100	10	51.0	197.1	1.2	0.2
UH-V_2_		2					50.5	201.6		
UH-V_3_		3					52.5	207.2		
UH-V_4_		4					48.5	185.1		

## Data Availability

The data presented in this study are available within the article.
